# Destabilization of the IFT-B cilia core complex due to mutations in IFT81 causes a Spectrum of Short-Rib Polydactyly Syndrome

**DOI:** 10.1038/srep34232

**Published:** 2016-09-26

**Authors:** Ivan Duran, S. Paige Taylor, Wenjuan Zhang, Jorge Martin, Kimberly N. Forlenza, Rhonda P. Spiro, Deborah A. Nickerson, Michael Bamshad, Daniel H. Cohn, Deborah Krakow

**Affiliations:** 1 Department of Orthopaedic Surgery, David Geffen School of Medicine at the University of California at Los Angeles, Los Angeles, California, 90095, USA; 2 Networking Biomedical Research Center in Bioengineering, Biomaterials and Nanomedicine, (CIBER-BBN), University of Malaga, Malaga, 29071, Spain; 3 Department of Human Genetics, David Geffen School of Medicine at the University of California at Los Angeles, Los Angeles, California, 90095, USA; 4 Department of Molecular, Cell, and Developmental Biology, University of California at Los Angeles, Los Angeles, California, 90095, USA; 5 Children’s Healthcare of Atlanta, Atlanta, GA, 30342, USA; 6 University of Washington Center for Mendelian Genomics, University of Washington, Seattle, Washington, 98195, USA; 7 Department of Obstetrics and Gynecology, David Geffen School of Medicine at the University of California at Los Angeles, Los Angeles, California, 90095, USA.

## Abstract

Short-rib polydactyly syndromes (SRPS) and Asphyxiating thoracic dystrophy (ATD) or Jeune Syndrome are recessively inherited skeletal ciliopathies characterized by profound skeletal abnormalities and are frequently associated with polydactyly and multiorgan system involvement. SRPS are produced by mutations in genes that participate in the formation and function of primary cilia and usually result from disruption of retrograde intraflagellar (IFT) transport of the cilium. Herein we describe a new spectrum of SRPS caused by mutations in the gene IFT81, a key component of the IFT-B complex essential for anterograde transport. In mutant chondrocytes, the mutations led to low levels of IFT81 and mutant cells produced elongated cilia, had altered hedgehog signaling, had increased post-translation modification of tubulin, and showed evidence of destabilization of additional anterograde transport complex components. These findings demonstrate the importance of IFT81 in the skeleton, its role in the anterograde transport complex, and expand the number of loci associated with SRPS.

Asphyxiating thoracic dystrophy (ATD) and the short rib polydactyly syndromes (SRPS) are autosomal recessively inherited skeletal disorders and are categorized as ciliopathies with major skeletal involvement^1^,^2^. Both are characterized by a long narrow chest that causes varying degree of respiratory distress, from minor insufficiency to respiratory failure and death. Skeletal features include short ribs, micromelia (shortened tubular bones), abnormal shaped roof of the acetabulum (trident-shaped) and frequently polydactyly. Non-skeletal features include retinal degeneration, and renal, pancreatic and liver abnormalities^3^,^4^. ATD can be milder and many individuals survive into young adulthood. Mutations in several genes have been associated with this phenotypic spectrum and include *DYNC2H1* [OMIM 603297], *DYNC2LI1, NEK1* [OMIM 604588], *IFT140* [OMIM 614620], *EVC1* [OMIM 604831], *EVC2* [OMIM 607261], *KIAA0586* [OMIM 610178], *CEP120* [OMIM 613446], *WDR19* [OMIM 608151]*, WDR34* [OMIM 613363]*, WDR35* [OMIM 613602]*, WDR60* [OMIM 615462]*, TTC21B* [OMIM 612014], *IFT172* [OMIM 607386] and *IFT80* [OMIM 611177]^5^,^6^,^7^,^8^,^9^,^10^,^11^,^12^,^13^,^14^,^15^,^16^,^17^,^18^,^19^,^20^. Many of the proteins encoded by the aforementioned genes participate in intraflagellar transport (IFT) in primary cilia, a sensory organelle present in most tissues and essential for specific signaling pathways. Ciliary structure and function depends on bidirectional transport (anterograde and retrograde) that mobilizes molecules from the base of the cilia to the tip and back. Each direction uses a different motor system for transport, kinesins for anterograde and dyneins for retrograde^21^,^22^,^23^. These motor systems bind IFT-B and IFT-A complexes, respectively, which mediate the intake and release of molecules into the cilia. Anterograde components of the IFT-B complex, IFT74 and IFT81 heterodimers are responsible for the binding of αβ-tubulin monomers during their transport to the tips of the cilia where they are released to polymerize and are key to microtubule-dependent ciliary function^24^,^25^,^26^,^27^. The IFT-B complex also carries retrograde components for later transport back to the base, and its disruption has been shown to cause abnormal ciliary distribution of tubulin and disrupted retrograde transport^28^. Intact cilia are necessary for Hedgehog (Hh) signaling^29^ and Hh ligands Sonic (SHH), Indian (IHH) and Desert (DHH) are highly involved in tissue specific cellular differentiation. Hedgehog ligands signal through their receptor Patched (PTCH) and Smoothened (SMO), which are localized to the cilia membrane. Upon binding of ligand to PTCH, repression of a second receptor, SMO, is released and SMO is activated and enters the cilium. Within the cilium, SMO regulates the activation of glioma-associated oncogene (GLI) transcription factors that control expression of Hedgehog (Hh) downstream targets^29^,^30^,^31^,^32^,^33^. Among the Hedgehog ligands, the Indian Hedgehog (IHH) signaling pathway is of particular importance in skeletal development. In the cartilage growth plate, IHH regulates the rate of hypertrophic differentiation, and alterations in this signaling cascade cause deleterious effects during skeletogenesis^34^,^35^,^36^,^37^. Abnormalities in cilia architecture and/or function affect Hedgehog signaling and contribute to the SRPS phenotype^4^,^5^.

## Results

### IFT81 mutations identified in SRPS cases

**Figure 1:**
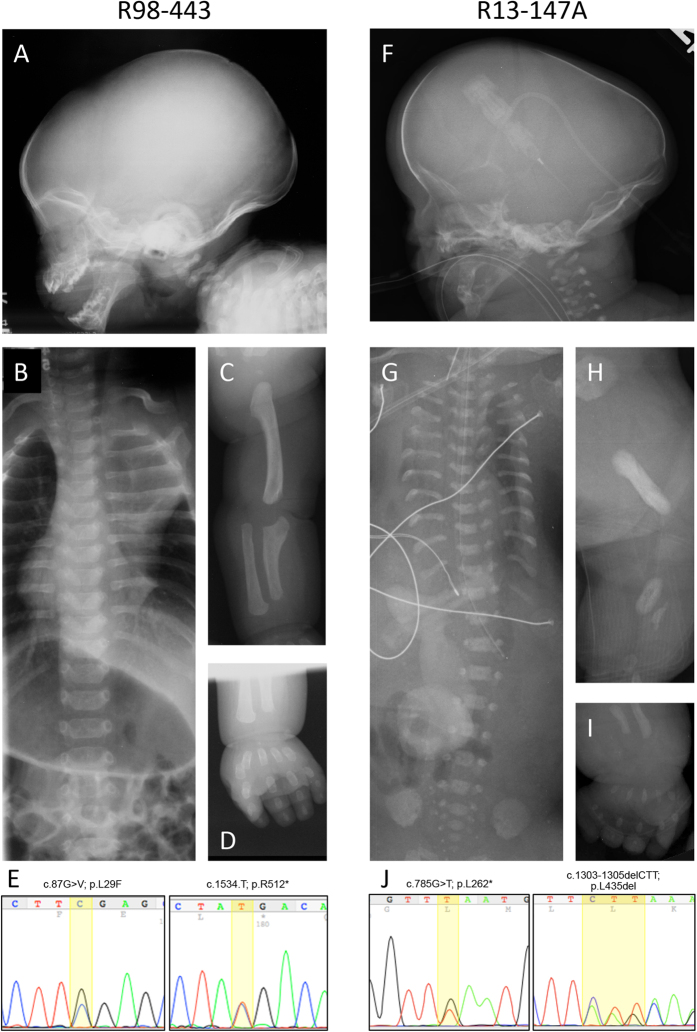
Mutations in IFT81 cause SRP syndrome. Radiographic findings in R98-443 and R13-147A. (**A,F**) show dolicocephaly, prominent occiput, and midface hypoplasia. (**B,G**) show long narrow thoraxes, handlebar clavicles in R98-443 and very short horizontal ribs in R13-147. (**C**) Upper extremity of R98-443 demonstrating shortened humerus and radius, and short, abnormally shaped ulna. (**H**) Lower extremity of R13-174 showing marked deficiency of the femur, tibia and fibula. (**D,I**) show brachydactyly and polydactyly and poor mineralization (arrow) in SRP case R13-174. (**E,J**) Chromatograms illustrating compound heterozygosity for mutations found in each case.

We ascertained a term male (International Skeletal Dysplasia Registry reference number R98-443) recognized at birth to have features consistent with ATD. The clinical findings are summarized in Table 1. The radiographic abnormalities included midface hypoplasia, dolichocephaly, a prominent occiput (Fig. 1A), short ribs, handlebar clavicles (Fig. 1B) and short, curved appendicular bones, with the upper limbs particularly abnormally shaped (Fig. 1C). There was no polydactyly on either the hands or feet (Fig. 1D). The infant developed respiratory distress soon after birth and was initially treated by supplemental oxygen. His respiratory compromised worsened over time and he died at 19 months of age.

We also ascertained a second case, (R13-147A), first suspected to have SRPS by prenatal ultrasonography. The fetus was delivered by cesarean section at 35 weeks of gestational age and died a few minutes after birth from respiratory failure. Postnatal radiographs showed dolichocephaly, a prominent occiput, midface hypoplasia (Fig. 1F), a very small thorax with shortened horizontal ribs (Fig. 1G), markedly short long bones with rounded metaphyses and marked hypoplasia of the radii, ulnae, tibiae and fibulae (Fig. 1H). Other radiographic features included small iliac bones and postaxial polydactyly of all extremities (Fig. 1I), consistent with a form of short rib polydactyly syndrome closely resembling SRPS type II or Mohr-Majewski syndrome^38^. Although at birth the genitalia appeared phenotypically female, karyotype analysis showed 46 XY, suggesting that the mutations led to sex reversal or ambiguous genitalia. Other clinical findings are noted in Table 1 and the presence and extent of multilevel organ involvement supports the subgroup of SRPS type II, Mohr-Majewski syndrome.

Exome sequence analyses identified variants in the ciliary gene that encodes IFT81 [OMIM 605489] in both cases. ATD case R98-443 showed compound heterozygosity for two variants: c.87G > C, predicting the protein change p.Leu29Phe (rs200335504_dbSNP) and c.1534C > T predicting the protein change p.Arg512* (rs200335504_dbSNP) (Fig. 1J). Both changes are of low allelic frequency (5.322e-05 and 1.659e-05, respectively), in the ExAC database (http://exac.broadinstitute.org). Leu29 is a highly evolutionarily conserved residue among vertebrates (Fig. S1) and the variant was rated as damaging by SIFT and PolyPhen with a MutationTaster prediction algorithm generating a probability of 0.999 by Bayes classifier (http://www.mutationtaster.org). The p.Arg512* mutation is predicted to be a null allele since the stop codon is not in the last or penultimate exon, so the transcript most likely undergoes nonsense-mediated decay (NMD).

The SRPS case, R13-147A, also showed compound heterozygosity for variants in *IFT81*: c.785T > G was predicted to cause a premature termination codon (p.Leu262*) and loss of the transcript. The second change, c.1303_1305delCTT, is predicted to result in an in-frame deletion of a highly evolutionarily conserved leucine residue (p.Leu435del) (Fig. 1J). Neither variant was found in the ExAC, 1000G or UW exome variant databases (http://www.1000genomes.org, http://evs.gs.washington.edu) and both were predicted to be damaging by MutationTaster with scores of 1 and 0.999, respectively. The Leu435 deletion is predicted to alter the third of four coiled-coil domains of ITF81, which could alter the conformation of the protein.

### IFT81 mutations destabilize anterograde IFT-B complex

**Figure 2:**
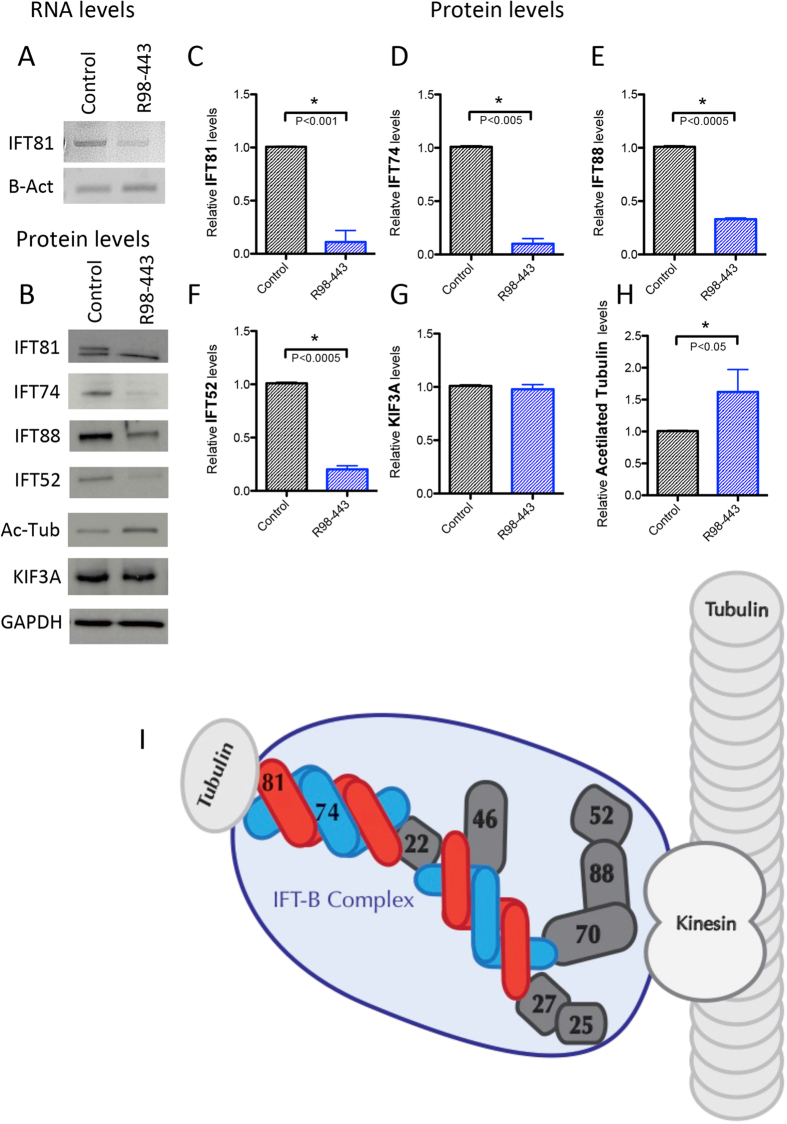
IFT-B complex is destabilized in IFT81 mutants. (**A**) RNA levels of IFT81 in control and patient chondrocytes. β-Actin serves as loading control. (**B**) Protein levels of several components of IFT-B complex (IFT81, IFT74, IFT88 and IFT52), acetyl-tubulin and kinesin motor component (KIF3A). GADPH serves as a loading control. (**C–H**) Bar graphs showing statistical analyses (t-Test) for the replicates of each studied protein between control and R98-443. (**I**) Cartoon of the IFT-B complex transporting αβ-tubulin through a cilia microtubule.

Cultured cells (chondrocytes) were only available for R98-443A and were used to assess the effect of the mutations on IFT81 mRNA and protein levels. RT-PCR demonstrated at least a 50 percent reduction in the transcript level in mutant cells relative to controls (Fig. 2A). Western blot analyses of IFT81 showed near complete loss of IFT81 in mutant cells (Fig. 2B). A non-specific band, that migrated slightly faster than IFT81, did not differ between control and mutant cells. These data demonstrate that compound heterozygosity for the mutations led to a significant loss of IFT81, more than could be accounted for by presence of one null allele, suggesting that the missense mutation destabilized IFT81.

In addition to the *IFT81* mutations, the exome sequence analysis in R98-443A identified heterozygosity for a known *TTC21B* variant (c.2600G > A; p.Arg867His; rs76726265). Because *TTC21B* mutations have shown to cause ATD, and this variant was predicted to be damaging (SIFT, PolyPhen with Bayes probability of 0.999 by the MutationTaster algorithm), mRNA and protein levels were characterized by RT-PCR and Western blot analysis respectively. The data (Fig. S2) showed no difference between case and control chondrocytes by either measure (Fig. S2). The allelic frequency for this variant was 0.0005 (ExAC), with 70 individuals showing heterozygosity for the allele. However, 64 of the 70 heterozygotes in the databases were African-American (ethnic-specific allelic frequency = 0.006), the same ethnicity as the proband, suggesting that the change is a polymorphism in that specific population.

IFT81 is a component of the IFT-B core complex that is responsible for the anterograde transport of tubulin and other molecules to the tip of the cilium. As diagrammed in Fig. 2I, the IFT-B core complex is composed of at least nine subunits 88, 81, 74, 70, 52, 46, 27, 25, and 22^24^,^39^. IFT74 directly interacts with IFT81 and heterodimers of IFT74/81 bind αβ-tubulin, regulating their transport into the cilia in a concentration dependent manner^25^,^26^,^27^. IFT52 is another key member of the core complex, and forms a trimeric complex with IFT70 and IFT88^40^,^41^,^42^. There is also a peripheral IFT-B complex composed of the IFT particles 20, 54, 57, 80, 172, and others. Based on the barely detectable levels of IFT81 protein resulting from the mutations, we tested the hypothesis that loss of IFT81 could lead to destabilization of the core IFT-B complex. Quantification of core complex proteins IFT74, IFT52 and IFT88 by Western blot analyses showed markedly decreased levels of all three proteins in R98-443A cells (Fig. 2B,D–F), demonstrating that mutations in IFT81 destabilized the IFT-B complex members at the protein level. To determine if mutated IFT81 also disrupted the stability of the kinesin motor responsible for the movement of the IFT-B complex, we analyzed protein levels of KIF3A. This key component of the kinesin complex^43^ showed similar protein levels between mutant and control cells (Fig. 2B,G), demonstrating that, although the anterograde IFT-B complex is altered, a key motor protein remained stable. As the IFT-B complex has been shown to bind tubulin monomers, we analyzed the levels of alpha-acetylated tubulin, a specific form of tubulin that constitutes the microtubules of cilia^44^,^45^. Acetylation of tubulin increases the stability of microtubules that comprise the cilium and recent data show that kinesin-1 prefers to travel on acetylated microtubule tracks^45^,^46^. We observed an increase of acetylated tubulin in R98-443 chondrocytes (Fig. 2B,H), suggesting impairment of proper turnover of tubulin in the cilium.

### IFT81 mutations alter ciliogenesis

**Figure 3:**
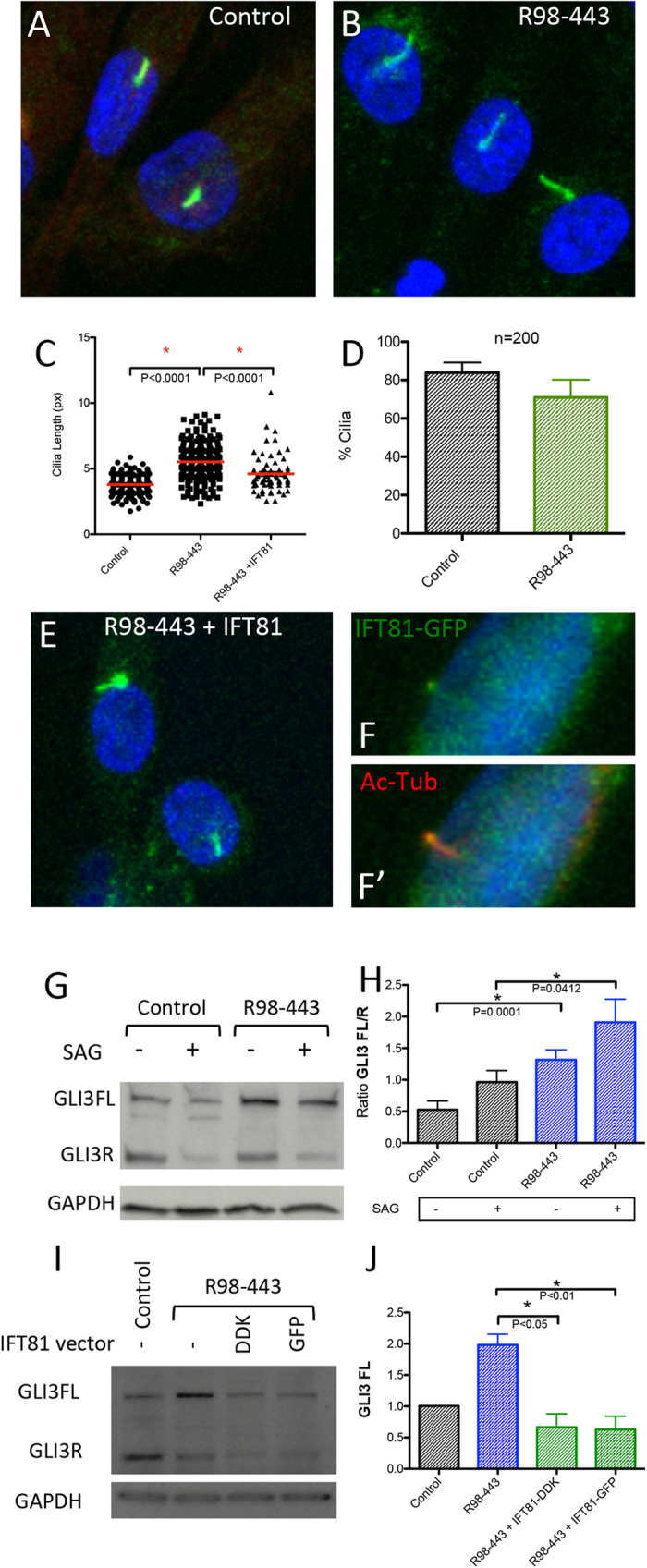
IFT81 mutations induce cilia defects and abnormal Hh signaling. (**A,B**) ARL13B and Pericentrin staining of the centrosome and cilia in green in control and R98-443 chondrocytes. (**C**) Cilia length of control, R98-443 and rescued R98-443 chondrocytes with IFT81 vector showing that rescued cells partially corrected cilia length phenotype. (**D**) Percentage of cells with cilia in control and patient chondrocytes showing no difference in number. (**E,F**) Cilia staining with ARL13B and Pericentrin (both green) in rescued R98-443 chondrocytes showing average length cilia. (**F,F’**) demonstrate that the IFT81-GFP fusion protein co-localized with Acetyl-Tubulin in the cilia. (**G,H**) GLI3 levels in control and R98-443 chondrocytes with and without SAG stimulation showing altered GLI3FL/R ratios. I-J GLI3FL levels were restored to control levels with R98-443 chondrocytes rescued with vectors of IFT81 fusion protein (GFP and DDK flag).

Although many of the mutations causing SRPS affect retrograde transport proteins, at a molecular level they disrupt the normal trafficking of molecules inside the cilia^5^,^15^,^47^,^48^. These alterations frequently lead to defects in ciliogenesis, cilia architecture and contribute to alter Hedgehog signaling activity. To investigate whether ciliogenesis was affected in mutant cells, we quantified both the abundance and length of cilia in mutant chondrocytes (Fig. 3A–D). The percentages of ciliated cells were similar between case and control cells (no difference detected by t-test p = 0.05) (Fig. 3D), but there was significant variability in cilia length among affected chondrocytes, with a significant increase in average cilia length in mutant cells (t-test P < 0.0001, Fig. 3C). To determine if expression of IFT81 could rescue the cilia phenotype, we expressed wild-type IFT81 (Origene) and again measured cilia length. As shown in Fig. 3, mutant cells transfected with wild-type IFT81 had reduced average cilia length compared with non-transfected cells, with cilia lengths similar to wild type cells.

### IFT81 mutations alter Hh signaling

To determine whether Hedgehog signaling was affected in mutant cells, we analyzed protein levels of GLI3, a bi-functional transcription factor that is activated through the Hh intracellular cascade. In the absence of Hh, GLI3 is proteolytically processed and acts as a transcriptional repressor (R) of Hh downstream targets. Conversely, binding of Hh ligands to the Patched receptor activates Smo in the cilia, leading to repression of the processing of GLI3, increased full-length (FL) GLI3 transcriptional activator and increased transcription of Hh downstream genes. In the normal state, the GLI3 FL/R ratio determines the transcriptional output of Hh signaling. In ATD chondrocytes there was an increased FL/R ratio at baseline (Fig. 3G,H) that was exaggerated in response to the Hh agonist SAG (Fig. 3G,H), implying IFT81 mutant cells have permanently dysregulated Hh signaling. Transfection of the cells with *IFT81* rescued the GLI3 FL levels (Fig. 3I,J) and partially rescued the FL/R ratio in mutant cells due to the simultaneous decrease of GLI3FL and R forms (Figs 3I and S3).

### Mutations in IFT81 disrupt growth plate

**Figure 4:**
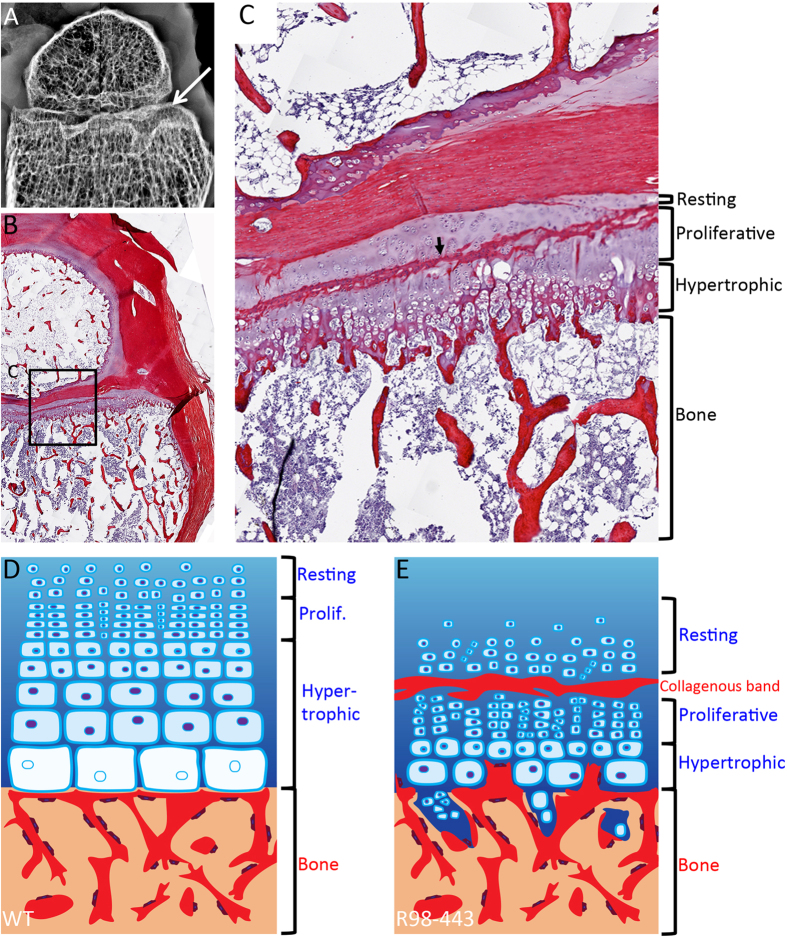
Growth plate defects in a patient with mutations in IFT81. (**A**) X-ray of formalin fixed distal femur at 19 months of age showing irregular metaphyseal margin (arrow). (**B,C**) Picrosirius Red-Haematoxylin staining of same distal femur (**B**) magnified in (**C**) showing irregular hypertrophic column formation and lack of normal progressive enlargement of hypertrophic chondrocytes. Arrow points to an irregular collagenous band transecting the growth plate. (**D,E**) Differences between control and IFT81 defective growth plates. (**D**) Control. (**E**) R98-443. Bone is represented in red and cartilage in blue.

While the data presented here demonstrate that IFT81 is required for normal cilia architecture and Hh signaling, how mutations in this gene affect skeletal morphogenesis remains unclear. Since most of the skeletal ciliopathies affect Hh signaling, and differentiation of growth plate chondrocytes is regulated in part by Indian Hedgehog (IHH), it is likely that altered IHH signaling due to defective cilia influences bone morphogenesis in SRPS^5^,^25^,^49^,^50^,^51^,^52^. To determine the involvement of mutations in IFT81 in this process, histologic analyses were performed using distal femur growth plates from case R98-443A (Fig. 4). Sections of growth plate were stained with picrosirius red and showed disorganization of chondrocytes in the proliferative zone, with short columns of cells dispersed in more than one plane (Fig. 4). There was a very short hypertrophic zone with poor column formation and lack of the normal progressive increasing size of hypertrophic chondrocytes as they approach the primary spongiosum (Fig. 4B,C). Interestingly, an unusual band of collagenous extracellular matrix with rounded cells was ectopically positioned in the middle of the proliferative zone and the staining was suggestive of a bone-like matrix (arrow in Fig. 4C).

## Discussion

Data presented here show that mutations in IFT81 destabilize the intraflagellar transport complex B, affect cilia architecture and Hh signaling, which results in altered growth plate and ultimately uncoupling the orderly process of skeletogenesis. However, differences in severity between the R98-443 and R13-147A phenotypes suggest that each mutation may exert different effects on functionality of the IFT81 protein. In both cases, one mutation is a null allele, so the phenotypic differences between the cases are likely to result from functional differences between the structural mutations. In the ATD case (R98-443), the missense mutation alters the calponin-like homology domain, which is involved in binding to the globular domain of αβ-tubulin and may interfere with this key interaction regulating cilia structure. The consequence of the in-frame deletion of a leucine at residue 435 in the SRPS case is less clear. This residue is in a coiled-coil domain (coiled-coil domain residues 416-456), which in IFT81 mediates interactions with IFT74 suggesting that the mutation may interfere with proper assembly of the IFT-B complex. The variant found in TTC21B gene in the R98-443 case could also act as a modifier of the phenotype^19^ although the high frequency of this variant in the population makes it unlikely.

To date, two other families with *IFT81* mutations have been described^53^. In both families the affected individuals had ciliopathy-like phenotypes. In one case there was homozygosity for a splice junction consensus sequence mutation predicted to lead to exon skipping and an in-frame deletion, implying loss of part of the second coiled-coil domain. The affected individual presented at birth with postaxial polydactyly of the feet, bilateral hyperechogenic kidneys and intellectual disability, with no reported retinal or skeletal findings. In the second case there was homozygosity for a small deletion toward the 3′ end of the gene, predicting a frameshift and extension of the IFT81 coding region beyond the normal stop codon. The affected individual showed retinal dystrophy and, over time, developed early-onset cone rod dystrophy. He was reported as having normal renal function and a normal skeleton. Phenotypically the cases reported here are quite distinct in that they primarily affect the skeleton, while the previously reported cases with *IFT81* mutations had no skeletal involvement beyond postaxial polydactyly seen in one case. Fibroblasts from the second case showed stable levels of IFT81 protein, and immunofluorescence for other IFT-B core complex components, specifically IFT25 and RABL5/IFT22, was not altered in the cells^53^. The fibroblasts had a reduced number of cilia that were shorter overall, findings that differed from what was observed in our study. Our ATD case showed no alteration in ciliogenesis, an increased average length of cilia, and destabilization of the IFT-B complex at the protein level. The mechanisms of disease in the skeletal ciliopathies studied here thus appear to be distinct. Destabilization of the IFT-B complex is accompanied by accumulation of acetylated-tubulin, the structural unit of the cilia microtubules. How this accumulation happens is unknown, but a possibility is that the destabilization of IFT-B complex leads to altered transport of acetylated-tubulin within the cilia. An emerging theme in the skeletal ciliopathies is that the ciliary abnormalities lead to loss of cilia length regulation^5^,^22^,^54^,^55^, a finding observed in the cases studied here. More specifically, longer cilia has been observed in other SRP cases with disruption of the IFT-B as well as other ciliopathies^5^,^22^,^56^,^57^. Studies have suggested that longer cilia are associated with increased anterograde IFT velocity and this can negatively impact the dynamic process of trafficking of signaling molecules and receptors into the ciliary compartment^58^. How this variability in length contributes to cellular and tissue abnormalities particularly in the skeleton remain unknown.

The abnormalities in Hh processing observed in the ATD mutant cells suggest that IHH signaling in the growth plate is likely to be altered. There are two aspects to the function of IHH in the growth plate that may be affected. IHH is synthesized by prehypertrophic and hypertrophic cells and regulates growth plate chondrocyte differentiation. The markedly abnormal structure of the ATD growth plate reflects defects in this process. In addition, IHH also determines the site of bone collar formation in the adjacent perichondrium, thus coupling chondrogenesis to osteogenesis during endochondral bone development^59^. The band of ectopic mineralized tissue across the growth plate suggests that inappropriate ossification effected through altered GLI3 FL/R ratios may have resulted from defective IFT81.

In conclusion, the data demonstrate that mutations in *IFT81* produce ATD and SRPS, and expand the number of genes associated with the skeletal ciliopathies. Markedly diminished levels of IFT81 led to instability of the IFT-B complex, loss of ciliary length regulation, altered post-translation modification of tubulin and abnormal Hh signaling. Through studies of the human skeletal ciliopathies, identification of mutations in the genes encoding each of the IFT particles have provided us with an integrated understanding of cilia function.

## Methods

Informed consent was obtained from all patients under an approved human subjects protocol. All experimental protocols and methods were carried out in accordance with institutional guidelines and regulations and approved by the UCLA Institutional Biosafety Committee.

### Exome analysis

DNA was isolated and submitted to the University of Washington Center for Mendelian Genomics for library preparation and exome sequencing in a cohort of patient with “ciliopathies with major skeletal involvement.” The samples were barcoded, captured using the NimbleGen SeqCap EZ Exome Library v2.0 probe library targeting 36.5Mb of genome, and sequenced on the Illumina GAIIx platform with 50 bp reads. Novoalign was used to align the sequencing data to the human reference genome [NCBI build 37] and the Genome Analysis Toolkit (GATK) was used for post-processing and variant calling according to GATK Best Practices recommendations. For each sample, at least 90% of targeted bases were covered by at least 10 independent reads. Variants were filtered against dbSNP137, 95 NIEHS EGP exome samples (v.0.0.8), 6503 exomes from the NHLBI Exome Sequencing Project (ESP6500), 1000 genomes (release 3.20120430), and 40 in-house exome samples. Mutations were further compared with known disease-causing mutations in HGMD (2012.2). Variants were annotated using VAX34 and mutation pathogenicity was predicted using the programs Polyphen35, Sift36, Condel37, and CADD38. Potential disease genes were identified under an autosomal recessive and X-linked recessive models, identifying either homozygous variants in one gene, two changes in the same gene, or hemizygous changes on the X chromosome because both of the potential for a hemizygous change producing disease. The changes were then filtered because on whether the genes had a known role in cilia and IFT81 was identified as known IFT-B cilia gene. All the variants identified by this strategy in the two cases are listed in Supplemental Table 1. The mutations reported in this work were confirmed by bidirectional Sanger sequencing of amplified DNA from the proband. 5′ 3′ primer sequences were IFT81 ex2 Fw ccaggacttttgttgccagt; IFT81 ex2 Rv ggctttctgctacccatcaa; IFT81 ex14 F ttctctgttgtttgggactgaa; and IFT81 ex14 Rv cacacctggcctgtatgtca. Sequence trace files were aligned and analyzed using 4peaks.

### Cell culture

Primary chondrocytes were isolated from distal femurs of affected individuals or age- matched normal controls by incubation of fragmented cartilage with 0.03% bacterial collagenase II. Primary chondrocytes were cultured in DMEM with 10% FBS in low passage number. Experiments were performed starving chondrocytes in 0.5% serum for 48 h previous to cell collection or SAG stimulation. SAG stimulation was performed by adding 500 nM SAG (R&D) in starving media for 24 h. Non-stimulated control cells were cultured 72 h in starvation to normalized cell growth.

### Western blot

Each antibody was tested in at least three experimental replicates from independent cultures. Cell lysates were collected from 80% confluence starved cultures in RIPA buffer with proteinase inhibitors (SIGMA). Lysates were cleared by centrifugation and quantified (Pierce BCA protein assay kit) to equal loading. 40 ug of each lysate were loaded with Laemmli buffer and run by 10% acrylamide SDS-PAGE gels and transfer to PVDF membranes. Membranes were saturated for 1 h and antibody-incubated overnight in 5% milk in TBST. HRP secondary antibody and ECL-film exposure was used to detect bands. Antibodies used were: IFT81 (Proteintech 1:1000); CMG or IFT74 (Abcam 1:500); IFT52 (Proteintech 1:1000); IFT88 (Proteintech 1:500); Aceto-Tubulin (SIGMA T6793 1:2000); KIF3A (Sigma 1:2000) Gli3 (R&D 1:200); GAPDH (Cell Signaling 1:1000). HRP secondary antibodies were Cell Signaling (1:1000, anti-mouse and–rabbit) and Santa Cruz (1:3000, anti-goat). FIJI was used to quantify bands following Gel Analysis recommendations from ImageJ and Gassmann *et al.* (http://rsb.info.nih.gov/ij/docs/menus/analyze.html#gels)^60^ and Mann-Whitney test was performed for statistic analysis using Prism software.

### RT-PCR

RNA from primary chondrocytes was extracted with Trizol and RNA was treated with DNase I (Invitrogen). Reverse transcription was performed using RevertAid First strand kit (Thermo). PCR was performed with Platinum Taq Polymerase. Coding sequence primers used were: IFT81 87 Fw: TCAATAAGGAGCCCTTTAGGAA; IFT81 87 Rv: AATCACCAAACCCTGACGAA; IFT81 1534 Fw: GAAAGGACGAACATTGGATGA; IFT81 1534 Rv: AAGACATTCTTCACGGAGTCTTC; Bact Fw: TCCCTGGAGAAGAGCTACGA; Bact Rv: AGGAAGGAAGGCTGGAAGAG.

### Immunofluorescence and Cilia measurements

Cells were cultured in 4-well chamber slides (LabTek). After serum starvation, cells were washed twice with PBS and fixed in PFA for 10 min and then permeabilized with 0.1% Triton-X-100 in PBS for 15 min at room temperature. Then cells were blocked in 10% goat serum for 1 hr at room temperature. Primary antibodies were diluted in PBS containing 1% serum and incubated overnight at 4 °C. Antibodies used were: ARL13B (Proteintech 1:100); anti-acetylated α-tubulin (Sigma T6793 1:2,000); Pericentrin (Abcam 1:2000); DAPI was used to stain nucleus. Detection was performed with secondary Alexa Fluor 488/568 antibodies (1:1000; Invitrogen). Images were captured on Zeiss Confocal 810. Images were collected with 1024 × 1024 pixel definition and Z-sections were taken at 0.5 μm step size. Max projections of the Z-stacks were used for primary cilium measurement and counting in ImageJ (NIH). Cilia length was measure in pixels (px) and compared from at least two experimental replicates with control chondrocytes (N = 226, 226, 67 cilia counted in control, R98-443, and rescued cells).

### Rescue experiment

Proband chondrocytes were electroporated with a vector containing CMV-IFT81 using a 4D-Nucleofector system and Amaxa P1 Kit under the program DS-150. Cells were recovered with starvation media and cultured for 48 h previous to cilia analysis.

### Histology

Distal femurs from the proband was collected after death at 6 months of age cut in half for better preservation and fixed in formalin 10%. Then, bone was decalcified in Immunocal (Formic Acid) for three days, dehydrated and embedded in paraffin. Sections of 10 um were obtained and stained with picrosirius red for 30 minutes and hematoxilline as counterstain.

## Additional Information

**How to cite this article**: Duran, I. *et al.* Destabilization of the IFT-B cilia core complex due to mutations in IFT81 causes a Spectrum of Short-Rib Polydactyly Syndrome. *Sci. Rep.* **6**, 34232; doi: 10.1038/srep34232 (2016).

## Supplementary Material

Supplementary Information

Supplementary Dataset S1

## Figures and Tables

**Table 1:** Clinical Findings.

Case	R98-443	R13-147A
Diagnosis	ATD	Short Rib Polydactyly Syndrome type II (Mohr-Majewski)
Gestational Age at Delivery (weeks)	Full term	35wk 4d
Birthweight (grams)	3797 g	3000 g
Birth Length (cm)	42.5 cm	38 cm
Apgar Scores	9, 9	Decreased minutes after birth
Head	Dolicocephaly, relative macrocephaly and with prominent occiput	Dolicocephaly, relative macrocephaly. Prominent forehead and occiput
Face	Prominent eyes, depressed nasal bridge with long philtrum	Flattened facies with midface hypoplasia
Ears	Right-sided pre-auricular pit without cystic swelling of the ear cartilage	Low set of ears
Heart	Normal	Ventricular septal defect
Thorax/Abdomen	Long and narrow thoracic cage with shortened ribs	Extreme shortened chest, severe pulmonary hypoplasia, omphalocele
Genitalia	Normal male. Both testes descended.	XY male with female genitalia
Neuro	Global hypotonia	Global hypotonia
Upper extremities	Rhizomelic shortening of the upper extremities greater than lower extremities, bilateral mesomelia of radii and ulnae	Extreme micromelia
Hands and feet	Broad hands and feet with short stubby fingers, bilateral simian creases and all of the fingers had two rather than three flexion creases	Polydactyly and syndactyly in the hands and feet
Lower extremities	Bowing of the mesomelic segment, dimples over the lateral knees	Extreme micromelia
