# Supplementary material for: Destabilization of the IFT-B cilia core complex due to mutations in IFT81 causes a Spectrum of Short-Rib Polydactyly Syndrome
Source: Sci Rep. 2016 Sep 26;6:34232. doi: 10.1038/srep34232 (PMC5035930; doi:10.1038/srep34232)
Supplement: Supplementary Information [file srep34232-s1.pdf]

Destabilization of the IFT-B cilia core complex due to mutations in IFT81 causes a Spectrum of Short-Rib Polydactyly Syndrome

Ivan Duran, S. Paige Taylor, Wenjuan Zhang, Jorge Martin, Kimberly N. Forlenza, Rhonda P. Spiro, Deborah A. Nickerson, Michael Bamshad, Daniel H. Cohn and Deborah Krakow*

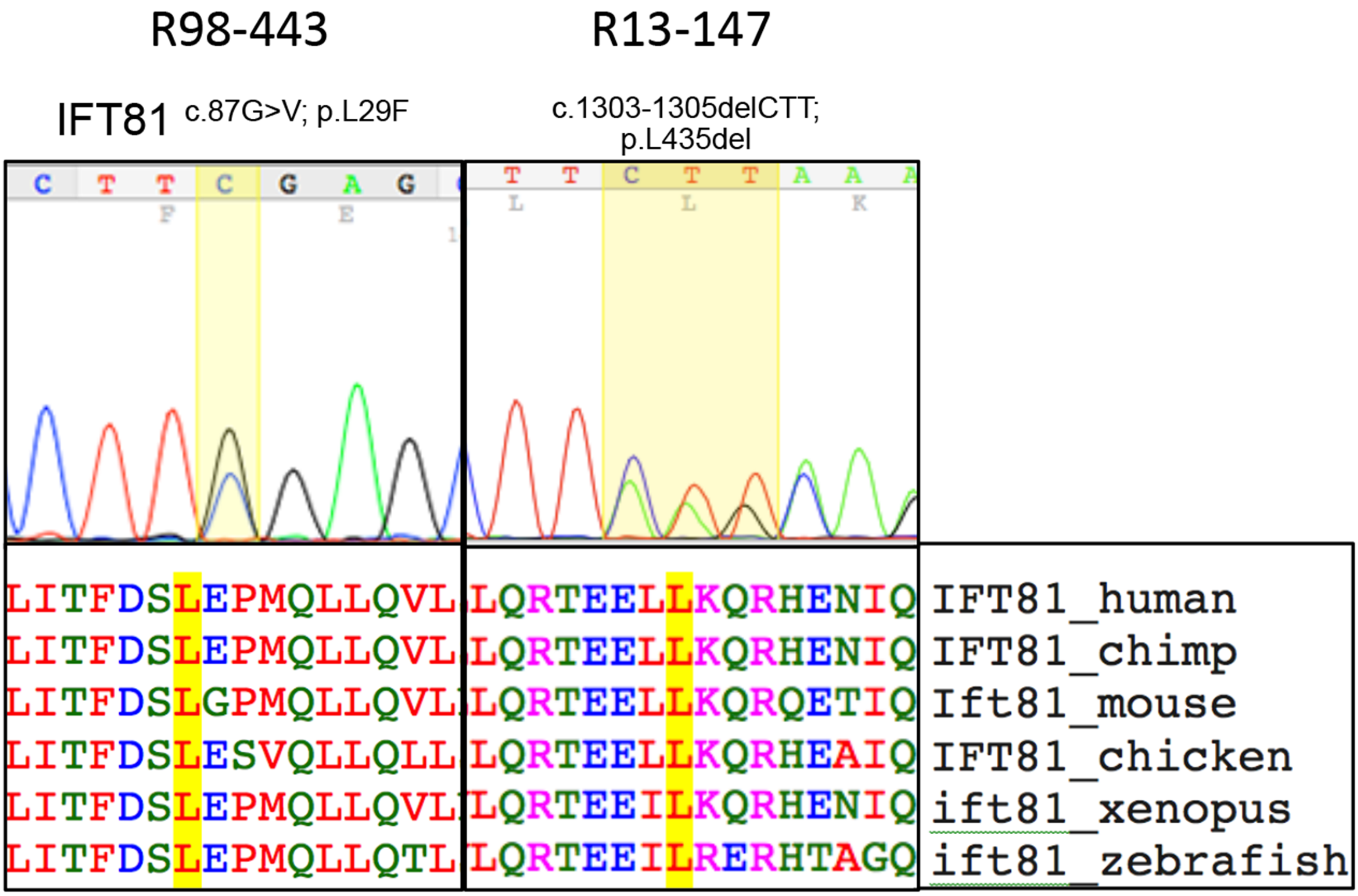

Supplemental Figure 1. Chromatograms demonstrating the missense and deletion mutations in R98-443 and R13-147 highlighting the conservation of the each respective residue across species.

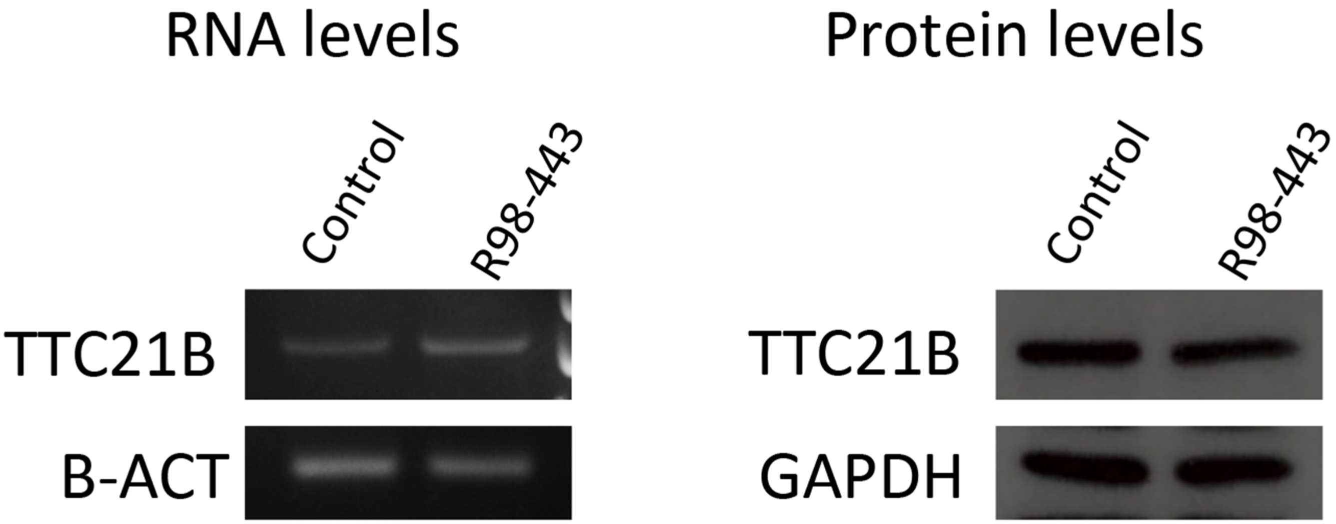

Supplemental Figure 2. RT-PCR and western blot analysis of TTC21B in R98-433 showing that the *TTC21B* variant (c.2600G>A; p.Arg867His; rs76726265) had no apparent effect on cDNA or protein levels in R98-443.

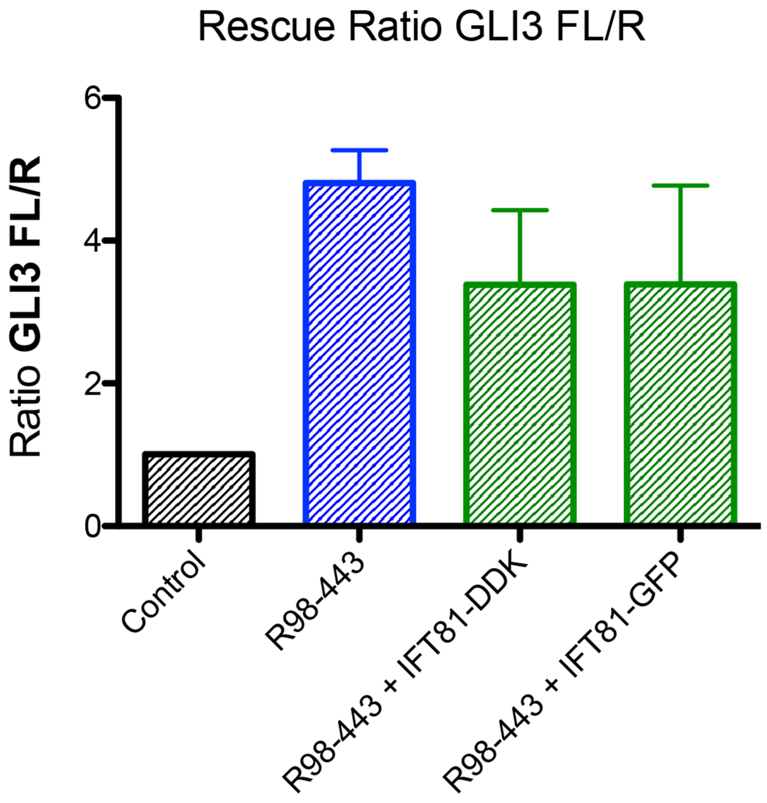

Supplemental Figure 3. Ratio of FL/R forms of GLI3 in R98-443 rescued with expression of wild type IFT81.
